# Hospital and Institutional News

**Published:** 1911-12-23

**Authors:** 


### HOSPITAL AND INSTITUTIONAL NEWS.

### SPALDING HOSPITAL PRODUCES ITS CHAMPION.

In the closest privacy, according to the local papers, the trustees of the Johnson Hospital, Spalding, continue to discuss Sir Henry Burdett's report, which appeared on November 18 (page 183), and was supplemented and enforced on December 2 (page 233). On the latter occasion point by point the trustees' brief "reply," though it had never been sent to us, was answered. In the meantime, however, the general silence is broken by Miss Tatam, who as "one of the staff" writes to the *Spalding Guardian* in defence of her beloved institution. The pertinence of her reply can be judged from the following remarkable statement: "I have lived with the matron over eight years and not one of us has ever known her tell an untruth." As nobody else has alluded to the matron the only suggestion that the matron has done so is thus implied by Miss Tatam herself. We mention this merely as showing how easily an inexperienced writer may trip herself when attempting the expert work of dealing with a criticism in detail. Miss Tatam simply denies the statements in the report, without the slightest attempt to argue or to disprove them in any way. "We are all very happy here" is the gist of her gentle refrain, but Sir Henry was careful to attribute to the staff "conscientious work notwithstanding the depressing surroundings."

### WHY THE RESERVE FUND IS NOT USED.

Miss Tatam naïvely suggests that the trustees are hoarding their large reserve in case a fire should break out! Perhaps nothing short of a fire would awaken the trustees to their responsibilities, but her simple-minded excuse for them is hardly one that they will be grateful for, since the laugh would be against them too badly if they offered it. On one point, however, Miss Tatam is definite. She "knows" that Sir Henry was asked to meet the trustees. As everyone knows, the writer of these reports for forty years has been regularly advising and meeting hospital committees. For the past two years indeed, in consequence of these reports he has been doing so more frequently than ever; it is obvious, therefore, that only a misapprehension could have prevented him from pointing out to the trustees in person the condition of their hospital. Miss Tatam deserves praise for her good intentions and becoming devotion, but she must bring rather heavier guns to bear the next time she wishes to make herself heard.

### A MEMORABLE MEETING.

The meeting of the League of Mercy at St. James' Palace on the 15th instant should prove of historic interest. King George and Queen Mary when Prince and Princess of Wales rendered many years of personal service through this League to the cause of the voluntary hospitals. No more striking proof of their continued interest could be given than the King Emperor's remembrance of the League's meeting in the midst of the absorbing and historical coronation ceremonies at Delhi. St. James's Palace with its historical attractions and most inviting and beautiful room of assembly, was a fitting place for this message from the King Emperor to be read to the members of the League of Mercy. The meeting proved to be probably the most successful and interesting one yet held. We hope arrangements may be made for this annual meeting of Presidents henceforth to assemble in St. James's Palace each year. The attendance was much larger than usual and the proceedings were graced by the presence of H.R.H. the Duchess of Albany and her Highness Princess Victoria of Schleswig-Holstein, both, like their Majesties, zealous and successful workers for the League. The chairman, Lord Farquhar, never spoke better, and the Hon. John Griffiths concluded the proceedings with an address, to be found in another column, which went straight to the hearts of all present. The audience was sent away full of pleasant memories and also merry hearted. This merry mood was due to a quaint remark of Mr. Griffiths, who, on rising to speak in St. James's Palace, said, "if it should be felt that he spoke with embarrassment it must be because looking at a picture of George III. for one hour was not the highest inspiration for eloquence." Annual meetings are often derided, but that on the 15th instant proves the possibility of making such gatherings, as we have often maintained, alive with continuous interest from start to finish.

### THE FIRST WOMAN FELLOW OF THE ROYAL COLLEGE OF SURGEONS.

The distinction of being the first woman to become a Fellow of the Royal College of Surgeons has fallen to Miss Eleanor Davies-Colley, who entered the London School of Medicine for Women, in connection with the Royal Free Hospital, in 1902. Miss Davies-Colley is an M.D. of London University, at which she graduated in 1910, having taken the degrees of M.B. and B.S. three years previously. It is interesting to add that Miss Davies-Colley's father had been a member of the College Council and formerly of the Court of Examiners. Miss Davies-Colley's appointments have included those of assistant anæsthetist at the Royal Free Hospital, senior house surgeon at the New Hospital for Women in Euston Road, and demonstrator in anatomy to the London School of Medicine for Women. The heartiest congratulations have been showered, not only on Miss Davies-Colley, but upon the London School of Medicine for Women, which since its first start under the hospitable shadow of the Royal Free Hospital has played the pioneer part in training women students who have won, one after another, the coveted medical distinctions that hitherto had remained beyond the reach of women's aspirations.

### A NEW SECRETARY FOR THE SATURDAY FUND.

Owing to the resignation of Mr. E. Radford of the post of organising secretary to the Hospital

Saturday Fund, Mr. W. H. Reed, the assistant organising secretary, has been promoted to succeed him. Mr. Radford, who has served the fund in the above capacity for the past five years, was chairman of the Woolwich Hospital Saturday local committee for eight years previously. He is leaving to take up the duties of assistant secretary to the Welsh National Memorial Fund to the late King, one object of which, as The Hospital has already stated, is the abolition of phthisis in Wales. It is interesting to note that Mr. Radford's resignation led to no fewer than 78 applications for his post. Let us add that the Lord Mayor, Sir Thomas Crosby, M.D., will be the President of the Saturday Fund during his year of office.

### HOSPITALS AND CHRISTMAS-BOXES.

The title of this paragraph will awaken in the minds of even the most generous institutional treasurers a sigh for the large sums which have to be disbursed at this season in tips. Those who receive them usually deserve them, but when money is scarce, as it certainly has been this year, such tips are a drain. The Gravesend Hospital, presumably prompted by Mr. Pearson, the Secretary, is ingeniously attempting to turn this old custom to its own advantage by circularising every householder with a letter signed by Lord Darnley, the President, and Mr. H. D. Stephenson, its Chairman, asking for a Christmas-box for the hospital from every house. This original idea, and gentle wrapping-up of an appeal, deserves to be recorded, as one which other provincial secretaries may like to adopt next year. The fact that people will give a Christmas box when they will refuse to subscribe to an appeal is one which is worth remembering.

### THE ART OF PERSUASION IN LEICESTER.

A proposal is being started to enlarge the Leicester Children's Hospital by converting the present third floor, which is disused, into a new ward of 20 beds, and by adding balconies and sanitary annexes to the floors below. The cost of this is expected to be £6,500, and to interest the public in the shilling fund which has been opened, a highly original pamphlet has been compiled. We have often congratulated Mr. Harry Johnson, the secretary of Leicester Infirmary, upon the capital manner in which his annual report is "got up" and illustrated every year; hardly less good is his latest piece of propaganda. The ingenious tag "there is no room in the Inn" associates the hospital's need for extension with Christmas sentiment very neatly, and if the series of photographs that follow is not as good as that with which the Report is usually embellished, it is quite good enough to induce anyone who sees the pamphlet to read between the illustrations. Every page of the cover has a sentence or two, including a quotation from Swinburne, that on the outside of the back cover being the best. We do not quote it, because hospital secretaries in want of an idea, would be well repaid by ordering their own copy.

### PROPOSED DAUGHTER HOSPITAL FOR OLDHAM INFIRMARY.

If you want to revenge yourself upon posterity perhaps the best way, as experience teaches, is to leave money for the foundation of a new institution, or for the endowment of a new need. To create new needs is the true function of philanthropy, but, as Dr. J. Kershaw's recent bequest to found a hospital at Royton, near Oldham, unfortunately shows, posterity does not always take kindly to the plan. Sufficient facts are not before us to decide whether Royton requires a new hospital for itself, or whether it is already served well enough by Oldham Infirmary. It is clear, however, that a medical man thought a new institution was needed, with the result that the District Council is now embarrassed with £40,000 which it does not know exactly how to spend. Apparently certain plans accompanied the bequest, whereby a hospital of thirty beds was projected, at a cost of £11,000, leaving £29,000 as an endowment fund. The District Council believes a further £1,100 above the income from this fund would be required for the annual upkeep of the institution, and apparently declines to be responsible for raising it. At the same time it considers it would be "ungenerous" to criticise—that is, in effect, to curtail—the plans. Dr. J. Kershaw's bequest has been left therefore to the tender mercies of a sub-committee, consisting of Councillors Smethhurst, H. Beswick, Brierley, and Gartside. Whatever is the result of their deliberations, we hope that this medical man's fine bequest will not be frittered away.

### EDINBURGH'S NEW PROFESSOR OF BACTERIOLOGY.

Who is going to fill the chair of Bacteriology about to be founded at Edinburgh University in pursuance of the late Mr. Robert Irvine's bequest? Several names are being offered, and the trustees, Dr. Sims Woodhead, Dr. David Patrick, Mr. William Maxwell, Mr. David Jerdan, and Mr. George Muir Wood are probably not sorry that this task is made to devolve upon the University, their own being ended with the handing over of the money.

### A "NEWTON" BED FOR DERBY INFIRMARY.

The former treasurer of the Derbyshire Royal Infirmary, the late Mr. Charles Edmund Newton, who held that office for 38 years, is to have a memorial to his memory through the generosity of his widow, the Hon. Mrs. Newton, who has given £1,000 to endow a bed in his name. Another endowed bed has been founded by Miss Louisa Bannerman, in memory of the late Miss Gertrude Louisa Newton.

### LONDON MENTAL HOSPITAL APPOINTMENTS.

Dr. M. B. Baines has resigned his appointment as fifth assistant medical officer at Claybury Mental Hospital, and the present sixth assistant, Mr. O. H. Bowen, has been appointed his successor. Mr. P. S. B. Langton has been appointed to fill the vacancy for sixth assistant medical officer thus to be caused. At Hanwell Mental Hospital Mr. Stanley Wyard has been appointed sixth assistant medical officer in succession to Mr. A. R. Littlejohn.

### LONDON'S NEW MENTAL HOSPITAL.

Hardly has the Asylums Committee of the London County Council proposed to provide a new mental hospital on the Horton Estate, when certain London Boards of Guardians have suggested that a new mental hospital is more necessary on the north, that is on the opposite side of the Thames. The Committee states that the unoccupied and very suitable site at Horton must settle the situation of the new hospital. This will provide for two thousand patients on the Horton Estate, and will be the eleventh mental hospital under the Council's control. It will probably cost about £509,000, though it is not yet determined whether the whole or only a part of the new hospital shall be built at once.

### THE COST OF RECURRENT INSANITY.

Pursuing the subject of recurrent insanity from another aspect, we note that the Finance Committee of the London County Council has been considering the total debt outstanding in respect of mental hospitals. This is £2,040,549, a reduction of some £38,000 as compared with the previous year. The number of patients in the county mental hospitals at the beginning of the year (including private patients at Claybury Hall) was 20,066, the possible accommodation available being for five more patients. The weekly cost of maintenance of each patient in the London mental hospitals, excluding private patients, for whom special rates are charged, averaged just over 10s. The average weekly cost varied from 9s. 8.84d. at Banstead to 10s. 7.25d. at Cane Hill, while at the Epileptic Colony, where the conditions are quite different, the cost was 13s. 5.44d. The average cost per week of patients sent to other asylums was 15s. The average weekly cost was below the weekly charge of 9s. 11d. a patient at only two of the mental hospitals—Banstead and Hanwell. The total expenditure of the Committee was £15,671 more than the receipts. These figures tell their own tale in the light of the appalling incidence of recurrent insanity which we considered last week. Remove that recurrency, whether by segregation or sterilisation, and half the expense would be curtailed in one year. Can anyone deny, moreover, that it is inevitably a wasteful expenditure? What is the experience of Mr. Spark, or of Dr. Robert Jones? In the meantime Mr. Walter Reynolds has given notice of motion that the Asylums Committee be instructed to report fully on the causes of recurrent insanity.

### WESTERN INFIRMARY, GLASGOW, AND RATE AID.

Lord Provost Stevenson, of Glasgow, took the extraordinary opportunity afforded him when presiding at the annual meeting of the Western Infirmary, of voicing his views as to the desirability of having the voluntary hospitals supported by the rates. He admitted that it required some courage to urge an opinion "which would very likely find very little sympathy in that room," and in the meantime, however, he said that the Infirmary's directors were fully justified in appealing to the generosity of the citizens. Turning then to the subject of the meeting, the Lord Provost noted that the deficiency of the general public's subscriptions was made up by those from the working classes, which showed a large increase. Sir Mathew Arthur pointed out that the Lord Provost was quite right in thinking that neither Sir Mathew nor any member of the Board was in sympathy with his suggestion that the voluntary principle should be abandoned.

### THE WEST END HOSPITAL'S NEW COMMITTEE

Lord Cardross and Mr. E. B. Hoare have recently joined the Committee of Management of the West End Hospital for Nervous Diseases, and the other day a number of ladies met at 5 Bryanston Square, on the invitation of Mrs. Ian Malcolm, one of the oldest supporters of the Hospital, to form a Ladies' Guild. Among the ladies present were Mrs. Audley Miles, Mrs. Harry Graham, the Countess of Dunmore, Mrs. Dundas Grant, Mrs. Ferdinand Huth, Miss Broadhurst, Lady Guernsey, Lady Sybil Grey, Lady Muriel Willoughby, and Mrs. Laming Evans. The chair was taken by Colonel the Hon. Charles S. H. D. Willoughby, Treasurer of the Hospital; the Secretary, Mr. D. D. Kirkaldy, and the Matron, Miss Catharine E. A. Thorpe, were also present. The following officers were elected: President, Lady Violet Brassey; Hon. Treasurer, Colonel Willoughby; Hon. Secretary, Miss Broadhurst; Hon. Secretary of Vegetable League, Lady Guernsey; Hon. Secretary of Clothing and Linen League, Miss Easton. Eventually a Committee was formed, consisting of Mrs. Ian Malcolm, Mrs. Ferdinand Huth, Mrs. Charles A. Ballance, Mrs. Harry Graham, and the Hon. Secretaries. Linen Leagues and Ladies' Guilds are sometimes supposed to be dull things, but hospital secretaries have learnt to respect them as among their most useful organisations.

### A HOSPITAL TREASURER ON SERVANTS AS IN-PATIENTS.

Sir John Barker, Bart., has had a pleasant task as treasurer of the Bishops Stortford Hospital in announcing that an adverse balance of £66 has been converted during the year into a £50 surplus. "While I am delighted at this," Sir John remarked, "as a successful business man I do not like to see the dropping off of subscriptions. We have a large number of servants using the hospital, and I think the Committee will have seriously to consider whether it should not accept them at 10s. a week unless they are in the service of subscribers." The word "accept" is equivocal, and those not present at the address can hardly tell whether it implies an increased or a decreased charge. If we may judge the latter from Sir John's implied sympathy with those servants, who, he said, often find on recovery that their places have been filled, well and good; if not, so astute a hospital officer and man of affairs will realise that any change of attitude just now on the part of hospitals towards in-patients who happen to be domestic servants would be hazardous in the extreme. On this point we should like to hear the opinions of the re-elected honorary secretaries, Mr. W. G. Gould and Mr. A. Boardman.

### A PROFESSOR ON ACTIVE SERVICE.

It is not often that the profession of arms recruits that of University study, but such was the case with Dr. Samuel McBean, late Professor of Materia Medica and Therapeutics at the University of Durham, who has died after a long illness at his residence in Newcastle at the age of seventy-two. Early in his career Dr. McBean was appointed to the Royal Naval Hospital, Plymouth, and received a commission as surgeon in the Royal Navy in 1862. He saw active service in the Far East, and was present at the bombardment of Kagosyma and of Simonoseki, Japan. He also acted as surgeon to the Royal Naval Hospital at Yokohama. Later he attended an expedition up the Niger on H.M.S. *Lee* in 1867. The following year Dr. McBean retired from the Navy and settled in Newcastle, where he built up an influential family practice. In the College of Medicine he successively held lectureships on botany, materia medica, and therapeutics, and in 1888 the University of Durham conferred upon him the honorary degree of M.A. Dr. McBean was held in high esteem by the students, and as an examiner he was noted for his kindly treatment of the candidates. He leaves six children, two sons being in the medical profession, of whom one continues the practice and the other is in the Royal Navy. The funeral was attended by a large number of the students of the Medical College and many of the professors.

### MANCHESTER CHAPLAINS' DERELICTION OF DUTY.

If one, and only one, example were required to show the lamentable falling off in the contributions of modern congregations on Hospital Sunday, the position in Manchester provides it. In announcing the large falling off which the Hospital Sunday Fund has this year to suffer, Mr. Charles Behrens, ex-Lord Mayor, startled his audience by confessing "the amount collected in the churches does not average £10 a church." It cannot be repeated too often that for this incredible indifference the clergy, and especially the hospital chaplains, must be held directly responsible.

### A NEW HOSPITAL FOR DUNDEE.

Most unexpectedly the Committee of the Private Hospital for Women in Dundee, which has been harassed for some time with the old problem how to adapt a building that really requires to be pulled down, has received £4,000 from Mrs. F. B. Sharp, St. Fort. This will be used for building a new hospital on a new site, and the £1,700 odd received from the annual bazaar will probably be set free for equipment purposes. The secretary, Miss Walker, has to admit a debt, but must be encouraged by Dr. Templeman's recent statement that "an institution of this kind which helps women who are willing to help themselves as far as their means permit," is to be commended. It will be seen, in short, that the private Hospital stands in a rather peculiar position, with a special function, that should benefit by a change of site. The new Committee with the responsibility for conducting the transfer includes Mr. William Mackenzie and Dr. Templeman as new members, while Miss Donald has been appointed assistant treasurer.

### PROGRESS AT HOLT SANATORIUM.

The long-expected date for laying the foundation stone of the children's sanatorium, Holt, Norfolk, arrived last Tuesday. As readers of The Hospital should remember, the children have been occupying temporary quarters for the most part, in which during five years the work has been carried on. As we pointed out in our issue of June 11, 1910, immense impetus was given to the sanatorium by the grant of £500 from King Edward's Hospital Fund. This was not merely an authoritative recognition and sign of approval, but also reminded the public of the large proportion of London children which was received at Holt. Mr. T. H. Beit, who performed the ceremony, is thus completing the organisation which Mr. Otto Beit's generosity began. Mr. Alfred Hoare, the treasurer, the Rev. E. C. Bedford, chairman, and Mr. T. H. Wyatt, M.V.O., the honorary secretary, are to be congratulated on Tuesday's ceremony, as marking a step in their consistent efforts not merely to erect buildings, but to secure an adequate maintenance for them when built.

### DOCTORS IN THE PULPIT ON HOSPITAL SUNDAY.

The attempt to stir up renewed interest in Sheffield on behalf of Hospital Sunday has produced the original suggestion that medical men should occupy the pulpits on that day. Instances of hospital secretaries performing this function are by no means unknown. Some, as members of the Free Churches, are not infrequently in the habit of addressing the local chapel-goers, while a few secretaries who are members of the Church of England are licensed lay preachers, and thus are entitled to occupy the lectern, if not the pulpit. A doctor at either post would be harder to find, and we think it better that the necessary renewal of enthusiasm should be the product of a genuine renewal of work on the part of hospital chaplains, who are directly responsible, than that a purely factitious interest should be excited by seeing medical men usurping their place. For the purpose of pleading the cause of the hospitals the institutional chaplain is by profession better equipped than the honorary medical staff, which can hardly be expected to undertake the duties of chaplains who have failed to act up to them, simply in order to tickle the public curiosity by attempting to give the pulpit an adventitious attraction on one Sunday in the year.

### ATTEMPTS TO REVIVE HOSPITAL SUNDAY IN SHEFFIELD.

It is only fair to say that the extraordinary failure of the Sheffield congregations to raise anything approaching a worthy contribution on Hospital Sunday has led to the beginnings of united action by all the religious bodies of the city. A deputation from these has waited upon the Hospital Sunday Committee, and, while proposals for altering the date of Hospital Sunday from January 21, any way will not affect its observance next month, a sub-committee has been appointed, consisting of Mr. E. Willoughby Firth and Dr. Porter, representing the Royal Infirmary; Mr. J. H. Doncaster and Mr. P. K. Wake, representing the Royal Hospital; Mr. G. Yeomans, representing the Jessop Hospital; and Mr. J. D. Webster, representing the Children's Hospital. Mr. J. W. Barnes, secretary, was also present. The result of the sub-committee's deliberations will be awaited with interest.

### THE RODERICK BEQUESTS TO BIRMINGHAM.

Not long ago the late Mr. Silver's bequests to three London Hospitals created a small sensation by their munificence, but a parallel was announced in Birmingham on Saturday last by the will of Mr. John Roderick, a well-known auctioneer. In it he bequeathed £50,000 to the General Hospital Birmingham, £10,000 to the Queen's Hospital, £1,000 each to the General Dispensary, and to the Skin and Urinary Hospital, while all these institutions except the Skin Hospital are to receive shares in his residuary estate. We trust that this bequest coinciding oddly with the passing of the Insurance Act will not be the last of its size, nor mark a change in the dispositions of rich people to support the voluntary system on a similar scale.

### A HOSPITAL PRESIDENT'S FINE EXAMPLE.

Owing to the generosity of Mr. John Sykes, J.P., President of the Huddersfield Infirmary, the debt on the building fund of £1,000 has been paid off, so as, in Mr. Sykes' own words, "we may be able to begin the New Year with a clean slate." The Board has passed a resolution thanking Mr. Sykes, who has set an admirable example this Christmas to all his hospital's subscribers, which all who realise the crisis involved by the passing of an unamended Insurance Act will appreciate at its full value.

### A LORD MAYOR'S SUCCESS AS COMMITTEEMAN.

It is worth while recording the attraction which hospital committee work has for the Lord Mayor of Cardiff, Alderman J. W. Courtis. His Lordship and the Lady Mayoress for many years have been generous supporters of King Edward VII.'s Hospital, Cardiff, and more than that have been active routine workers. Alderman Courtis preceded Colonel Bruce Vaughan as Chairman of the House Committee, and Mrs. Courtis is a useful member of that and other committees, so that their new appeal is based upon personal knowledge of hospital needs. The Lord Mayor calls for a generous response to the anonymous challenger who has offered to contribute £500 if a further sum of £4,500 be subscribed by Christmas. Of this, as The Hospital previously reported, Lord Merthyr promised £500, and a like sum comes from another anonymous friend. The Lord Mayor, however, takes up the challenge afresh and has inspirited everyone by saying, "My wife and I will have pleasure in adding our names for another £500, so there now remains only £3,000 to be raised by Christmas." At the present time nearly 1,000 patients are waiting admission, of whom over 300 are children. A wealthy city like Cardiff should take a warning from our Special Appeal Number of last week, and realise how to escape the example of Mr. Thomas Scrooge by generously raising the required £3,000 in time for the result to be announced from the pulpits on Christmas Day.

### MODERN SURGERY IN RELATION TO SERIOUS CRIME.

When passing sentence at the close of a recent case in Edinburgh, the judge commented upon the relation that modern surgery may bear towards the crime with which a prisoner may be charged. After receiving three bullet-wounds in the head, the wife of the man convicted of shooting her had recovered. The judge indicated that but for the skilled treatment of modern surgery the charge would have been that of murder. The good fortune of the prisoner, who probably long ere he was brought before the bar of justice had regretted an act committed under the "short madness" of anger, it can hardly be disputed, rests upon the introduction of antiseptic surgery. There is a pathetic, not to say a gruesome, illustration of this in a provincial medical museum. A surgeon of the old days amongst other tastes had leanings towards anything that savoured of the dreadful. For example, he carried off in high glee, through the streets of his native city, partly hidden under a cloak, the remains of the gibbet irons in which an unfortunate murderer had been suspended.

### A GRUESOME STORY.

This surgeon had occasion to trephine the skull of an unfortunate girl who had received a blow on the head from a stone thrown at her by an angry lover. Trephining under surgical conditions of a hundred years ago we should probably consider now to be more likely to render the injured person the victim of the surgeon than of the stone-thrower. However that may be, the girl died, and as a consequence of her death the young man suffered the death penalty. The surgeon—whom we cannot suspect for a moment as having had any qualms of conscience regarding his share in the serious termination—has preserved an account of all the circumstances of the case, even to pathetic details of the man's attitude towards death in the last few moments of his life, in a book bound in the murderer's own skin. In justice to the surgeon it should, however, be mentioned that his feelings compelled him to turn away from the final details of the scene.

### AN APPEAL IN A HUNDRED.

The interest in the Paddington Green Children's Hospital of Mr. George Hanbury, whose name will always be associated with the origin and development of this institution, shows no signs of flagging. Hardly is the work of adapting, building, and opening the new out-patient department over than Mr. Hanbury is at it again appealing for £1,877 to pay all the cost of this extension. We cordially commend this appeal—which we hope will be met by the end of the year—to all those who care for the progressive efficiency of children's hospitals. If only to reward the work of such veterans in personal service to the sick as Mr. W. H. Pearce and Mr. George Hanbury, the money should be given before Christmas Day.

### THE MACDONALD MEMORIAL AT LEICESTER.

The many friends of the late Mrs. Macdonald, the wife of Mr. J. Ramsay Macdonald, present Chairman of the Labour Party, have combined to perpetuate her memory and to show their appreciation of the large amount of social work she accomplished during her lifetime, by founding some appropriate memorial. After some discussion it has been decided that the memorial is to take the form of a piece of sculpture embodying "an idea that might clearly show her character and ideals." In addition the committee appeals for subscriptions to two other memorials, which may possibly interest a wider section of the public to whom Mrs. Macdonald's activity in all matters concerning the health of the nation is well-known. One of these is the Macdonald-Middleton Baby Clinic which was recently opened at 12 Telford Road, North Kensington. The clinic is in charge of Dr. Ethel Bentham and among the consulting staff is Sir Victor Horsley. A resident fully trained nurse carries out whatever treatment may be ordered, and helps the mothers in various ways. The importance of such a baby clinic has long been realised, and the Kensington establishment deserves a visit from everyone who is interested in child welfare and the problem of inculcating the elementary principles of the hygiene of childhood upon the community. The third object which the memorial committee has in view is the endowing of a new ward in the Leicester Children's Hospital.

### WHY LEICESTER INFIRMARY WAS CHOSEN.

Since the time when her husband became the junior member for Leicester, Mrs. Macdonald took the keenest and most active interest in the health work of the town, and her sympathy with and personal service in aid of, the infirmary, of which the Children's Hospital forms an integral part, were well known and cordially appreciated by the authorities of this institution. The Leicester Infirmary can pride itself on the fact that the working men of its district take a practical interest in its activities. They contribute annually a sum of over £9,000 towards its funds, and they are adequately represented on the board of management. It is therefore particularly appropriate that this splendid institution should have been selected by the memorial committee as the special one to be associated with the memorial. The chairman of the local committee is Mr. C. J. Bond, F.R.C.S.; the treasurer is Sir Edward Wood, and the secretary Mr. Harry H. Peach. We may mention that the treasurer of the Baby Clinic at Kensington is Mrs. P. Nodin, of Minock, Kenley, Surrey. Contributions to the memorial fund may be sent to any of the above gentlemen or to Mrs. D'Arcy Hart, who is the treasurer of the memorial executive committee for London.

### UNSTANDARDISED SCRIPT WRITING.

Probably it never occurs to medical men that differences in prescribing may cause confusion and thus prevent the good dispensing they require for their patients. Some prescribers are in the habit of writing their prescriptions for the full quantity of medicine required, such as a six or an eight ounce bottleful, whilst others write for one dose and add instructions to the dispenser how many doses are to be given. If each physician strictly adhered to one of these two methods no complaint could possibly be made, but this is not always done, as sometimes the prescriber may adopt both methods. For example, a man may prescribe thus: ℞ Liquor Strychnin. ♏vi. Aq. ad. ℥i., this being for a single dose. On the other hand, the following may be written: ℞ Liquor Trinitrini ♏vi. Aq. ad ℥vi., thus giving the total quantity required and not the actual dose. It is these varieties of method, especially when dealing with drugs which are not in everyday use, which may mislead the busy dispenser.

### THIS WEEK'S DRUG MARKET.

A quieter tone prevails in the drug market and for the remainder of the year business is not likely to be active. A number of alterations in prices have been made, some of which are of importance. Rectified spirit, methylated spirit and ethers are slightly dearer. Carbolic acid has been advanced in price to the extent of 15 per cent., and the tendency is still in an upward direction. Makers of codeine, as was expected, have announced a further advance in price; opium and morphine are unchanged, but the tendency of prices is upwards. Apomorphine is dearer. Gallic acid, tannic acid, saffron and almond oil are also dearer. Lower prices are expected for glycerine and it would be advisable to refrain from purchasing more than sufficient for immediate requirements.

### TO OUR READERS.

Owing to the exceptional pressure on our space at Christmas time the continuation of Dr. Alfred Norman's article on "Electricity in Modern Medicine" is unavoidably held over from this week.

